# Extrusion puffing pretreated cereals for rapid production of high-maltose syrup

**DOI:** 10.1016/j.fochx.2022.100445

**Published:** 2022-09-16

**Authors:** Hung-I Chien, Yung-Hsiang Tsai, Hui-Min David Wang, Cheng-Di Dong, Chun-Yung Huang, Chia-Hung Kuo

**Affiliations:** a Department of Seafood Science, National Kaohsiung University of Science and Technology, 142 Haijhuan Road, Nan-Tzu District, Kaohsiung 811, Taiwan; b Institute of Aquatic Science and Technology, National Kaohsiung University of Science and Technology, Kaohsiung, Taiwan; c Graduate Institute of Biomedical Engineering, National Chung Hsing University, Taichung 402, Taiwan; d Department of Marine Environmental Engineering, National Kaohsiung University of Science and Technology, Kaohsiung 811, Taiwan; e Center for Aquatic Products Inspection Service, National Kaohsiung University of Science and Technology, Kaohsiung 811, Taiwan

**Keywords:** Amylase, Cereals, Extrusion puffing, Gelatinization, Maltose syrup, Starch

## Abstract

In this study, cereals with high starch content, including brown rice, corn, and buckwheat were pretreated by extrusion. The physicochemical properties of extruded-puffed cereals obtained from different extrusion conditions were analyzed herein. The puffed extrudates exhibited lower bulk density, higher water solubility and gelatinization as compared to untreated cereals. The FTIR-ATR results confirmed a decrease in the crystalline structure of extruded-puffed cereals. A higher V_max_/K_m_ value was observed in the enzymatic saccharification of puffed extrudates that significantly improved hydrolysis rate and yield. Finally, the high-maltose syrup was produced via the enzymatic hydrolysis of extruded-puffed cereals at high substrate concentrations (20 %). After hydrolysis for 180 min at an enzyme substrate ratio (E/S ratio) of 0.2, the syrup with dextrose equivalent (DE) value of 63, 62, and 61 were obtained from extruded-puffed brown rice, corn, and buckwheat, respectively. Our results showed the potential of using extruded-puffed cereals for producing high-maltose syrup.

## Highlights

• Extrusion puffing of cereals improved their water solubility and gelatinization.
• FTIR-ATR study revealed structural differences between native and puffed cereals.
• Extrusion puffing highly enhanced the efficiency of saccharification.
• The extruded-puffed cereals had a higher V_max_/K_m_ value as compared to native.
• Extruded-puffed cereals showed potential for high-maltose syrup production.

## Graphical abstract

**Figure:**
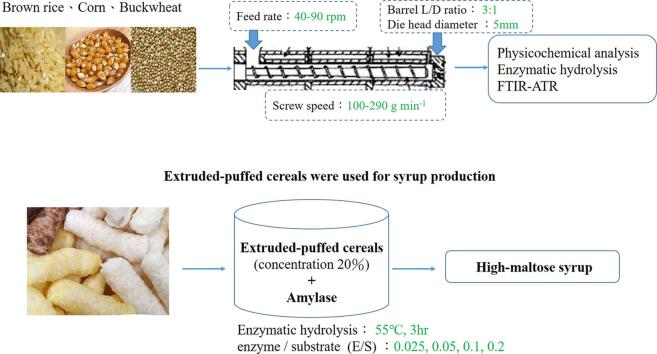

## 1 Introduction

Extrusion puffing is a food processing technology commonly used for treatment of agricultural products; it offers the advantages of diversified products, continuous and low labor-intensive production, high yield and no pollution. Extrusion puffing is commonly used in the food industry for processing cereals or crops with high starch content, such as rice and corn, into puffed cereal chips or puffed snacks (Paznocht et al., 2021, Stojceska et al., 2009). Extrusion has been used to change the physical and chemical structure of biomass to improve its digestibility during enzymatic hydrolysis (Zheng & Rehmann, 2014). The extrusion of Douglas-fir wood at lower feed moisture and extrusion temperatures increased fermentable sugar production during enzymatic hydrolysis (Gu, Wolcott, & Ganjyal, 2018). Similarly, the enzymatic hydrolysis of extruded soybean hulls increased the glucose yield by 132 % as compared to untreated biomass (Yoo, Alavi, Vadlani, & Amanor-Boadu, 2011).

The extrusion puffing process combines several unit operations: heating, shearing, crushing and mixing, to create a high temperature and pressure environment in a specific barrel. The cereals in the barrel are gelatinized and then extruded through a die. The internal moisture of melted cereals is instantly evaporated, thereby forming a pressure difference, and resulting in the cereal expanding with a porous structure and the starch gelatinized (Li et al., 2019, Moraru and Kokini, 2003). Starch is the most abundant ingredient in cereals and the most important ingredient that provides energy sources for animals and plants. The changes in the physical and chemical properties of cereals after extrusion have been discussed. Vanier et al. (2016) found that the expansion ratio is related to the amylose content, with higher amylose having a lower expansion ratio. Extrusion cooking of chickpea increases starch and protein digestibility and soluble dietary fiber content in the extrudates (Yağcı, Altan, & Doğan, 2020). The extrusion process provides heat and shear forces to restructure the starch into irregular chunks or flakes, thereby reducing its overall density and promoting the rapid penetration of water into the puffed extrudates (Roberts et al., 2014, Wang et al., 2020).

Any starchy crops, such as corn, rice, cassava and potatoes, can be used to produce maltose syrup. The production of starch syrup first gelatinizes starch powder and then acid or enzyme is used to hydrolyze it into dextrose, maltose, and dextrins. The maltose syrup with dextrose equivalent (DE) of 50–70, is called high conversion syrup, and mainly contains a high amount of maltose and a small amount of glucose and dextrins. Amylases are biochemical catalysts with specificity to hydrolyze starch. α-Amylase acts on both amylose and amylopectin, and indiscriminately hydrolyses α-1,4 glycosidic linkages in starch. β-Amylase successively cleaves the α-1,4-glucan chain in units of maltose from the non-reducing end to produce maltose. The molecular structure of amylose is linear, while that of amylopectin is highly branched (Fan et al., 2021). For unbranched substrates like amylose, it can be completely hydrolyzed to yield maltose and a small amount of glucose. However, amylase acts on amylopectin or glucan; the hydrolysis reaction stops at the front of α-1,6-glycosidic linkage, so a limit dextrin with a relatively large molecular weight is generated. With cereals under high temperature, high pressure and high shear force in the extruder, the hydrogen bonds between starch molecules are disrupted, the starch is gelatinized, and the water solubility of starch increases. Starch in extrudates becomes more accessible as a result of macromolecular degradation caused by extrusion (Barbiroli, Bonomi, Casiraghi, Iametti, Pagani, & Marti, 2013). After the subsequent action by amylase, the hydrolysis yield of extruded starch can be greatly improved. These advantages show that the extrusion technology to pretreat crops with high starch content can be applied to the rapid saccharification of starch. Therefore, extrusion was used to pretreat cereals instead of traditional cooking, and the feasibility of using extruded cereals for rapid saccharification to produce maltose syrup was attempted herein.

In this study, three crops: brown rice, corn, and buckwheat were selected to study the effect of extrusion feed speed and screw speed on the physicochemical properties of puffed extrudates. The physicochemical properties of puffed extrudates were analyzed, including moisture, bulk density, water solubility index, expansion ratio and degree of gelatinization. The structure changes of native and puffed extrudate were determined by Fourier transform infrared spectroscopy with attenuated total reflection (FTIR- ATR). The effect of extrusion on the enzymatic hydrolysis enhancement was evaluated through the enzymatic kinetic model. Finally, the feasibility of using puffed extrudates as raw material for producing maltose syrup was explored.

## 2 Materials and methods

### 2.1 Materials

Brown rice (9.2 % moisture, 71.3 % carbohydrate, 7.9 % protein and 2.4 % lipid) was purchased from Golden Rice Castle Co., ltd. (Taitung, Taiwan). Corn (9.3 % moisture, 71.7 % carbohydrate, 10.9 % protein and 3.9 % lipid) and buckwheat (9.3 % moisture, 79.8 % carbohydrate, 7.2 % protein and 0.8 % lipid) were purchased from Wang Laichang Enterprise Co., ltd. (Kaohsiung, Taiwan). Food-grade amylase (starch saccharification activity 600 U g^−1^; one starch saccharifying unit is defined as the amount of enzyme that catalyzes an increase in reducing activity equivalent to 1 mg of glucose per minute) was purchased from Amano Enzyme Inc. (Nagoya, Japan). All other chemicals were analytical grade and commercially available.

### 2.2 Extrusion experiments

The machine used for this experiment was a single-screw extruder (C1 Type, Yuan Chuang Food Machinery Co. ltd., Taiwan) equipped with a 5 mm diameter die head. The barrel diameter and L/D ratio were 35 mm and 3:1, respectively. The actual operating temperature of the barrel was 140 °C. The whole grains were fed directly into the extruder for treatment. The screw speed was set at 55–95 rpm. The feed rate was set at 130 to 290 g min^−1^, 100–220 g min^−1^, and 115–265 g min^−1^ for brown rice, corn, and buckwheat, respectively. The cereals were fed directly to the single-screw extruder, and the extrudates from the die head were cut by a four-leaf blade at a speed of 10 rpm. Specific mechanical energy (SMEs; J g^-1^) was calculated based on the following equation:

(1) $$SME=\frac{2\pi w\tau_{0}}{M}$$

where w is the screw speed (rpm), τ_o_ is the corrected torque (N ⋅ m), and M is the feed rate (g min^−1^).

### 2.3 Physicochemical analysis

The bulk densities of the native materials and extrudates samples were measured using the volumetric displacement method as described by Hwang and Yakawa (1980), and 0.1 mm glass beads were used as a displacement medium. The moisture content was measured using a halogen moisture analyzer (SH10A, Shanghai Jinghai Instrument Co., ltd.). The expansion ratio, expressing the expansion of the extrudate, was calculated by the ratio between the average diameter of the extrudates and the diameter of the extruder die. An average of 30 samples was used for each extrusion condition. The water solubility index (WSI) and degree of gelatinization based on the amylose/iodine blue value were determined according to the methods described previously (Kuo et al., 2019).

### 2.4 FTIR-ATR analysis

The FTIR spectra were collected using a Thermo Scientific Nicolet iS5 FT-IR spectrometer with an iD5 single bounce ATR accessory equipped with a ZnSe crystal. The samples were scanned for each spectrum; 64 scans were recorded at a resolution of 4 cm^−1^ in a range of 4000 cm^−1^ to 600 cm^−1^.

### 2.5 Enzymatic hydrolysis of extrudates

A 0.5 g (dry basis) sample was suspended in 25 ml of 100 mM phosphate buffer (pH 5). Amylase concentration of 120 U g^−1^ substrate was used in the hydrolysis reaction. The hydrolysis was carried out in an orbital shaking water bath at 55 °C and 200 rpm for 3 h. The release of soluble reducing sugars was periodically measured by 3,5-dinitrosalicylic acid (DNS) assay and calculated by subtracting the reducing sugar content of the blank of the amylase solution (Kuo, Shieh, Huang, Wang, & Huang, 2019).

### 2.6 Kinetics of enzymatic hydrolysis

0.1 g of amylase (60 U) was added to 25 ml of 100 mM phosphate buffer (pH 5) at substrate concentrations of 5, 10, 15, and 20 g/L. Enzymatic hydrolysis was carried through in an orbital shaking water bath at 55 °C and 200 rpm for 10 min. In order to determine the initial rate of enzymatic hydrolysis, the reducing sugar was measured, expressed as grams of reducing sugar released per liter per minute. The initial rates of each substrate concentration were used to draw Lineweaver-Burk plots. The kinetic parameters (V_max_ and K_m_) were calculated according to the Lineweaver-Burk equation as follows:

(2) $$\frac{1}{v_{0}}=\left(\frac{K_{m}}{V_{\max}}\right)\left(\frac{1}{S_{0}}\right)+\frac{1}{V_{\max}}$$

where S_0_ is initial concentration of substrate; v_0_ is initial rate of reaction; K_m_ is Michaelis-Menten constant; V_max_ is maximum rate of reaction.

### 2.7 Production of high-maltose syrup

The extrudates were added with water to a substrate concentration of 20 % (w/v) for production of maltose syrup, and amylase was added to the above solution to adjust the enzyme substrate ratio (E/S, w/w) at 0.025, 0.05, 0.1, and 0.2, respectively. Enzymatic saccharification was carried at 55 °C for 5 h. Samples were taken at different times to measure the reducing sugar content by DNS method. Dextrose equivalent (DE) is defined as the amount of reducing sugars in syrup as a percentage of dry original solid weight, and is calculated as follows:

DE value = (reducing sugar weight / original solid weight) × 100 % (3).

### 2.8 Sugar content of maltose syrup

Sugar content of maltose syrup was determined by an HPLC system, consisting of a Hitachi L-2130 HPLC pump and a Hitachi L-2490 refractive index detector (Hitachi, Tokyo, Japan). The amino column with high acetonitrile eluents was used, to analyze sugar (Kuo et al., 2004, Li et al., 2016). A 250 mm × 4.6 mm Hypersil APS-2 column (Thermo Instrument Systems Inc., Runcorn, UK) was employed and the mobile phase consisted of acetonitrile and distilled water (82/18) at a flow rate of 1 ml min^−1^.

### 2.9 Statistical analysis

JMP software (SAS Institute Inc., Cary, NC, USA) was used to analyze the experimental data using one-way analysis of variance (AVOVA). The student's test was applied to compare each group's means; significance was defined at P < 0.05.

## 3 Results and discussion

### 3.1 Effects of extrusion parameters on the properties of expanded-puffed brown rice, corn and buckwheat

This study used extrusion to pretreat three common cereals: brown rice, corn and buckwheat, and their extrudates were used as raw material for the production of maltose syrup. The brown rice, corn and buckwheat were each fed directly into the extruder to investigate the effect of the operating parameters of the extruder, screw speed and feed rate on the physiochemical properties of each extruded product. Table 1 presents the moisture, bulk density, expansion ratio, WSI, gelatinization degree and specific mechanical energy of the extruded products. After extrusion, the moisture content of extruded cereals tends to decrease. As compared to the native cereals, the moisture of extruded brown rice, corn, and buckwheat was decreased by 1.79–2.76 %, 1.44–2.1 % and 1.67–3.5 %, respectively. The results showed that the extruded cereals have different levels of water loss. When the feed rate was fixed, changing the screw speed had less effect on the moisture. However, the expanded cereals' moisture increased with the increase in the feed rate when the screw speed was fixed because increasing the feed rate decreased the residence time of the cereal in the barrel, resulting in the extruded-puffed cereals having higher moisture. The results indicated that the retention time of the cereals in the barrel had a higher effect on moisture than the screw speed. The bulk density of native brown rice, corn, and buckwheat was 715.88, 989.88, and 788.83 kg m^−3^, respectively. The bulk density is considered as an index of the puffing extent. After extrusion, the bulk density of extruded brown rice, corn, and buckwheat was decreased to 85.91–108.75, 83.10–120.83, and 588.48–667.49 kg m^−3^, respectively. The bulk density of extruded cereals decreased with the increased screw speed at a fixed feed rate but increased with an increasing feed rate at a fixed screw speed. The lowest bulk density was 85.91, 83.10, and 588.48 kg m^−3^ for brown rice, corn and buckwheat, respectively, and their extrusion conditions were screw speed of 95 rpm and feed rates of 130, 100, and 115 g min^−1^, respectively. In general, effective puffed cereals have a bulk density around 80 ∼ 140 g min^−1^ (Kantrong, Charunuch, Limsangouan, & Pengpinit, 2018). However, the bulk density of extruded buckwheat was higher than the other two cereals, indicating that buckwheat puffing required more machine power. Although extruded buckwheat had a higher overall density, its gelatinization degree was not much different from the other two cereals. Overall, the bulk density of extruded cereals was lower than that of native and cooked samples, indicating that the surface area of extruded cereals had increased. The expansion ratio increased with the screw speed from 55 to 95 rpm, and the largest expansion ratio was observed at a screw speed of 95 rpm and an appropriate feed speed around 100–130 g min^−1^. The expansion ratio is inversely proportional to density, which agrees with previous work (Berrios, Wood, Whitehand, & Pan, 2004). The water solubility index (WSI) is an indicator that reflects the degradation of starch, which is the amount of soluble components released from the extruded cereals (Yağcı & Göğüş, 2008). The WSI was 0.87 % and 1.55 % for native and cooked brown rice and was 0.15 % and 0.52 % for native and cooked corn, respectively. After extrusion, the WSI of extruded brown rice and corn increased significantly to 23.20–29.99 % and 10.65–17.19 %, respectively. However, the WSI was only 2.47–4.46 % for extruded buckwheat. The extruded buckwheat exhibited a lower WSI probably due to the high bulk density and low expansion ratio; as a result, the extruded buckwheat was relatively rigid and water could not easily penetrate to dissolve the soluble fraction in a limited time. Extrusion cooking has been used to increase the water solubility of sugar beet pulp (Rouilly, Jorda, & Rigal, 2006). Mechanical shear force and screw speed show the effect of the interaction of the two parameters on WSI. When the feed rate is fixed, the mechanical shear force increases with screw speed, which is consistent with the dependence of SME on screw speed as described in Equation (1). The WSI of the extrudates increased with increasing mechanical shear force. However, increasing the feed rate resulted in a decrease in WSI because reducing residence time of cereals in the barrel reduced heating and shearing time to cereals. The compressional puffing pretreatment has been shown to increase the extraction yield of fucoidan and chitosan (Huang, Kuo, & Lee, 2018). Gelatinization is the conversion of raw starch to a cooked and digestible material via the application of water and heat. Gelatinization is the major transition of starch in the crystalline state during thermal processes. The native cereals have a tightly packed crystalline starch structure. The moisture of the tightly packed crystalline starch was evaporated by the high temperature during the extrusion process, resulting in an environment of high temperature and high pressure formed in the barrel; the extrudates were then abruptly depressurized at the die to a normal pressure and the internal moisture rapidly evaporated. The extruded cereals were expanded to form a porous structure with a multiple flat layer. This represented that crystallized starch was transformed into an amorphous form during the extrusion. As shown in Table 1, the degree of gelatinization of extruded cereals was higher than that of the native and cooked cereals after extrusion. Our experimental results showed that the degree of gelatinization was proportional to the specific mechanical energy (SME). The SME is proportional to the screw speed and inversely proportional to the feed speed. Therefore, the highest degree of gelatinization was obtained to be 1.04, 1.03, and 1.03 for brown rice, corn, and buckwheat, under the maximum screw speed of 95 rpm and the feed speed of 130 g min^−1^, 100 g min^−1^, and 115 g min^−1^, respectively. Sun et al.'s (2019) experiments also found that increasing SME can reduce the crystallinity of extruded whole buckwheat noodles. In this extrusion experiment, the maximum SME were 45, 59, and 51 J g^−1^ for brown rice, corn, and buckwheat, respectively. SME is related to both feed speed and screw speed. The maximum SME indicated that the extruder was operated at a higher screw speed and a lower feed rate. The shear force formed by the high screw speed caused more damage to the starch granule of the cereals. At the low feed rate, the cereals were fully heated and gelatinized in the barrel. Therefore, the extrudates obtained at highest SME had the lowest moisture, lowest bulk density, highest WSI, and highest gelatinization.

**Table 1:** The effect of extrusion parameters on the physiochemical properties of extruded-puffed cereals.

Sample		Extrusion condition		Moisture (%)	Bulk density (kg m^-3^)		WSI	Gelatinization	SME**
		Screw speed (rpm)	Feed rate (g min^-1^)			Expansion ratio	(%)	degree	(J g^-1^)
Brown rice	Native	-	-	9.35±0.11^b*^	715.88±20.81^a^	-	0.87±0.10^c^	0.53 ±0.007^f^	-

	Cooked	-	-	45.67±0.78^a^	694.12±24.68^a^	-	1.55±0.48^c^	0.75±0.005^e^	-

	Extruded-puffed	55	130	6.59±0.05^e^	108.75±5.33^b^	2.73±0.09^c^	24.71±1.55^b^	0.93 ±0.0004^b^	26
		75	130	6.89±0.07^de^	92.15±1.69^bc^	2.77±0.12^c^	24.53±3.03^b^	0.93 ±0.006^b^	35
		95	130	6.71±0.14^de^	85.91±3.79^c^	3.09±0.12^b^	29.99±1.84^a^	1.04 ±0.007^a^	45
95		210	7.25±0.07^cd^	98.53±4.92^bc^	3.35±0.13^a^	29.64±1.96^a^	0.91 ±0.005^c^	28
95		290	7.56±0.26^c^	105.24±5.12^bc^	3.08±0.13^b^	23.20±4.33^b^	0.85 ±0.006^d^	20

Corn	Native	-	-	8.25±0.14^b^	989.88±60.55^a^	-	0.15±0.05^d^	0.61 ±0.004^f^	-

	Cooked	-	-	20.22±0.31^a^	881.07±8.59^b^	-	0.52±0.14^d^	0.71 ±0.007^e^	-

	Extruded-puffed	55	100	6.48±0.07^d^	120.83±0.31^c^	2.56±0.11^c^	10.65±0.92^c^	0.89 ±0.008^c^	34
		75	100	6.66±0.13^d^	112.76±5.45^c^	2.40±0.12^d^	11.17±1.65^c^	0.94 ±0.004^b^	46
		95	100	6.42±0.08^d^	83.10±4.59^c^	2.59±0.11^c^	17.19±0.42^a^	1.03 ±0.0002^a^	59
95		160	6.61±0.14^d^	97.76±1.65^c^	2.81±0.13^b^	15.52±1.28^b^	0.93 ±0.0004^b^	36
95		220	7.08±0.10^c^	102.68±5.04^c^	2.97±0.11^a^	15.88±0.31^ab^	0.85 ±0.007^d^	26

Buckwheat	Native	-	-	9.31±0.19^b^	788.83±14.37^a^	-	0.77±0.44^c^	0.46 ±0.02^g^	-

	Cooked	-	-	48.67±0.61^a^	599.41±17.17^c^	-	1.84±0.41^b^	0.68 ±0.008^f^	-

	Extruded-puffed	55	115	6.38±0.07^e^	610.84±8.37^c^	1.37±0.04^bc^	2.47±0.52^b^	0.86 ±0.0007^c^	29
		75	115	6.51±0.1^de^	592.97±2.70^c^	1.36±0.03^c^	2.62±0.51^b^	0.89 ±0.009^b^	40
		95	115	5.81±0.1^f^	588.48±25.40^c^	1.39±0.03^b^	3.92±3.60^a^	1.03 ±0.01^a^	51
95		190	6.95±0.07^d^	613.50±12.15^c^	1.43±0.05^a^	4.46±0.70^a^	0.82 ±0.007^d^	31
95		265	7.64±0.1^c^	667.49±21.32^b^	1.39±0.05^b^	3.65±0.52^a^	0.77 ±0.01^e^	22

* The samples of the same cereal with different letters indicate a significant difference at p < 0.05, according to the LSD (least significant difference) test.

** Specific mechanical energy (SME) was calculated using Equation (1).

### 3.2 FTIR-ATR measurement

FTIR-ATR is sensitive to the changes in the crystalline arrangement of starch granules. Especially, the spectrum of gelatinized starch is sensitive in the region of 1100–900 cm^−1^. The absorbance in this region has been used to reflect the crystalline fraction in the starches of native brown rice and brown rice extrudate (Kuo, Shieh, Huang, Wang, & Huang, 2019). Native starches that exhibit more ordered structure have been found to be more resistant to enzymatic hydrolysis (Sevenou, Hill, Farhat, & Mitchell, 2002). The gelatinization degree and crystallinity of starches have a considerable influence on the enzymatic saccharification. Starch granules absorb heat during gelatinization, resulting in disruption of the crystalline structure of the granules and loss of ordered structure. Therefore, FTIR-ATR can be used to measure the structural differences of starch molecules, and some characteristic absorption peaks are used as the infrared crystallinity index (Flores-Morales, Jiménez-Estrada, & Mora-Escobedo, 2012).

**Fig. 1:**
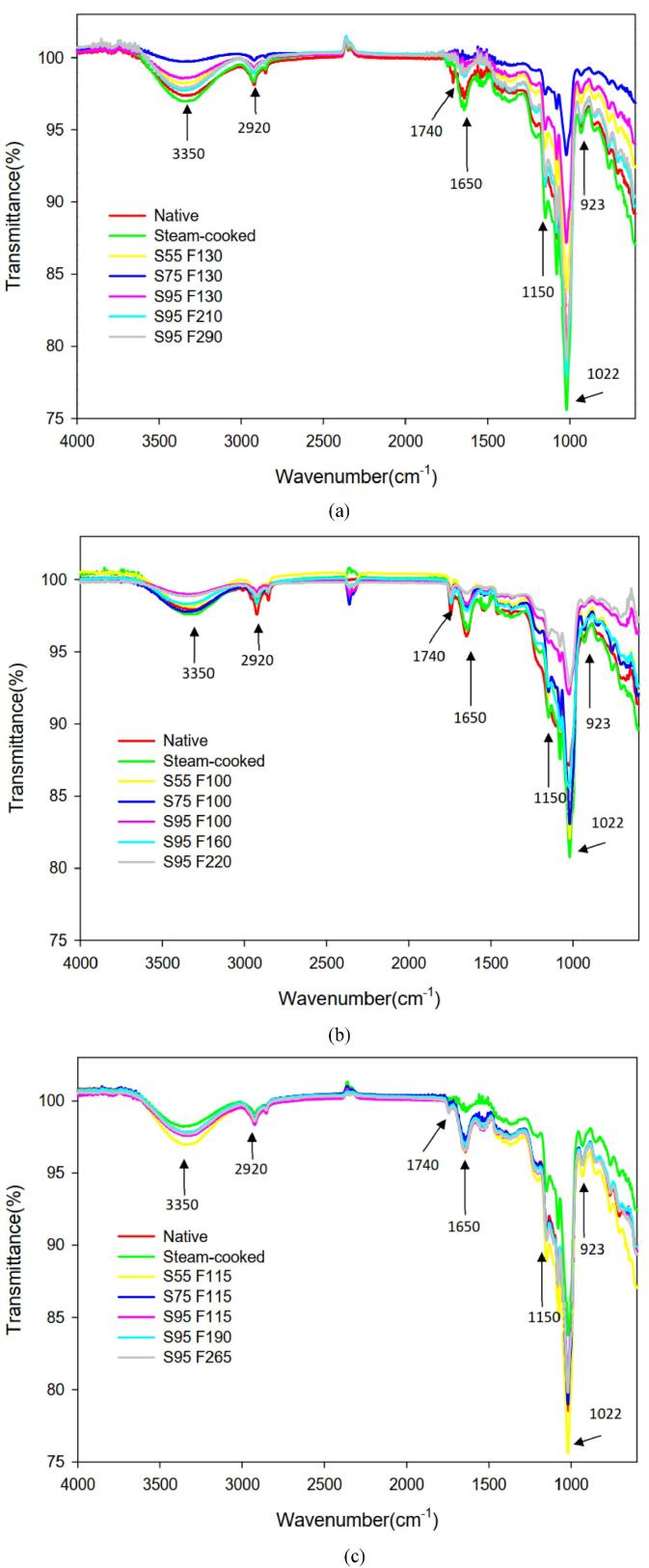
FTIR spectra of native, steam-cooked and extruded-puffed (a) brown rice (b) corn and (c) buckwheat. S and F represent the screw speed (rpm) and feed rate (g/min) of the extruder, respectively.

Fig. 1 shows FTIR spectra in the 4000–600 cm^−1^ wave number range for native, steam-cooked and extruded-puffed cereals. The hydroxyl group shows stretching vibration at 3350 cm^−1^. The peak at 2920 cm^−1^ is assigned to the C—H stretching vibration. The lipid and protein molecules present in the starch are showed C=O stretching vibration at 1740 and 1650 cm^−1^. The peaks in the region of 1150–900 cm^−1^ have shown to be sensitive to the changes in starch structure to the C—O stretching vibration; in particular bands at 995, 1022 and 1045 cm^−1^ have been shown to be sensitive to characterizing the ordered structure of starches. The absorbance bands at 995, 1022, and 1045 cm^−1^ are quite sensitive, which are associated to the ordered structure of starches and amorphous regions in starch. The absorption band at 1022 cm^−1^ is associated with the amorphous region of starches, and at 995 cm^−1^ and 1045 cm^−1^ are related to the crystalline regions of starch (Warren et al., 2011, Wei et al., 2011). The absorbance ratio at A1045/A1022 or 995/A1022 has been used to reflect the crystalline fraction in the starches (Kuo et al., 2019, Zhao et al., 2022). The absorption ratios of A1045/A1022 and A995/A1022 are shown in Table 2. The higher absorbance ratio of native cereals indicates that they had higher crystallinity and ordered structure. After cooking or extrudate, the A1045/A1022 of the brown rice, corn and buckwheat decreased from 0.76 to 0.66–0.71, 0.96 to 0.68–0.9 and 0.73 to 0.68–0.73, respectively. Similarly, the A995/A1022 of the brown rice, corn and buckwheat decreased from 0.77 to 0.69–0.72, 0.79 to 0.69–0.75 and 0.75 to 0.71–0.73, respectively. Our results showed that the absorbance ratios at A1045/A1022 and A995/A1022 decreased after cooking or extrusion, indicating that the starches changed their crystal structure from an ordered state to an amorphous state. Extrusion and cooking can effectively gelatinize starch, and the change in crystallinity observed by FTIR has the similar effect.

**Table 2:** The selected FTIR-ATR peak ratios for native, steam-cooked and extruded-puffed of cereals obtained from different extrusion conditions.

	Sample	Extrusion condition		FTIR-ATR	
		Screw Speed (rpm)	Feed Rate (g/min)	A1045/A1022 absorbance ratio	A995/A1022 absorbance ratio
Brown rice	Native	–	–	0.76	0.77

	Cooked	–	–	0.71	0.71

	Extruded-puffed	55	130	0.68	0.72
		75	130	0.67	0.69
		95	130	0.68	0.72
		95	210	0.67	0.72
		95	290	0.66	0.72

Corn	Native	–	–	0.96	0.79

	Cooked	–	–	0.74	0.72

		55	100	0.69	0.69
		75	100	0.68	0.71
	Extruded-puffed	95	100	0.88	0.71
		95	160	0.9	0.7
		95	220	0.74	0.75

Buckwheat	Native	–	–	0.73	0.75

	Cooked	–	–	0.68	0.73

		55	115	0.71	0.71
		75	115	0.71	0.72
	Extruded-puffed	95	115	0.73	0.72
		95	190	0.72	0.72
		95	265	0.72	0.71

### 3.3 Hydrolysis characteristics of extruded cereals by amylase

**Fig. 2:**
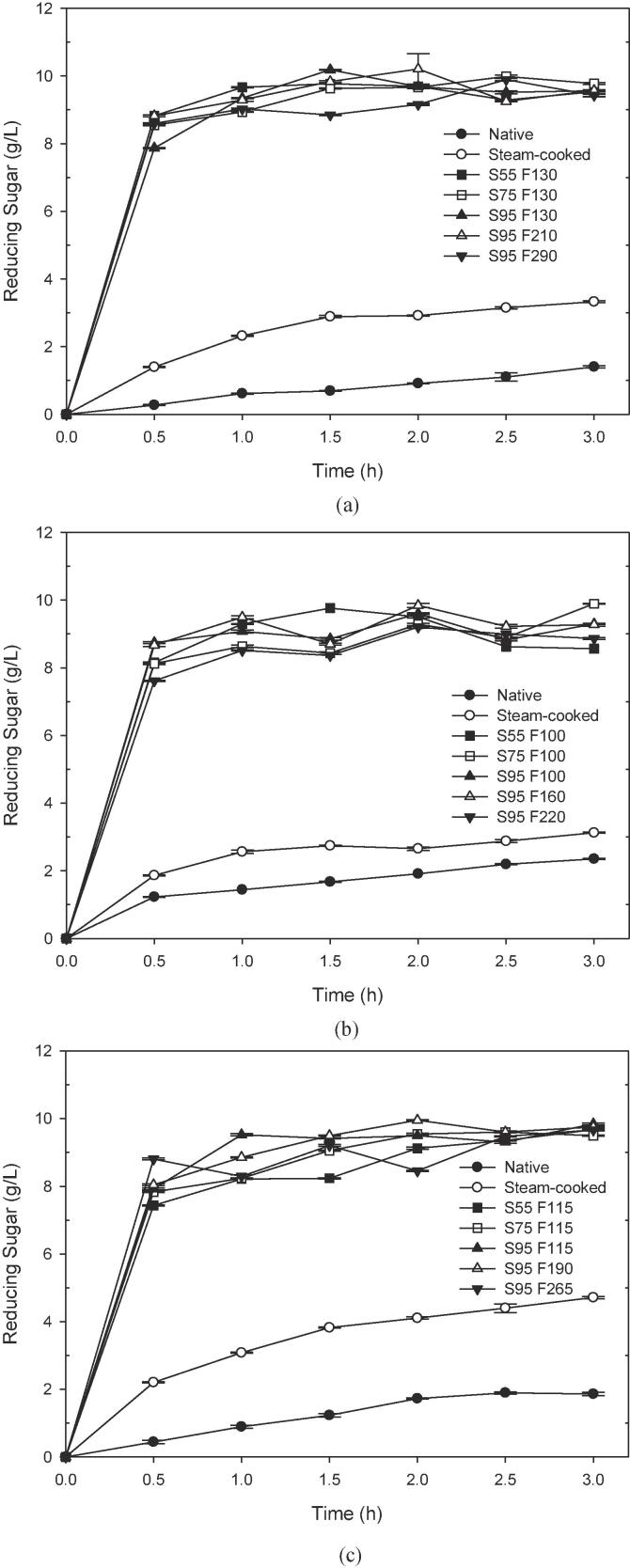
Reducing sugars released during enzymatic hydrolysis of native, steam-cooked and extruded-puffed (a) brown rice, (b) corn and (c) buckwheat. Hydrolysis conditions: substrate (dry basis) concentration of 20 g/L, amylase concentration of 120 U g^−1^ substrate and temperature of 55 °C. S and F represent the screw speed (rpm) and feed rate (g/min) of the extruder, respectively.

The extruded-puffed brown rice, corn and buckwheat obtained from various extrusion conditions were subjected to enzymatic hydrolysis. The total amount of reducing sugar released during the enzymatic hydrolysis of native, steam-cooked and extruded-puffed cereals is shown in Fig. 2. After amylase hydrolysis for 30 min, only 0.3, 1.2, and 0.4 g/L of reducing sugars were released from native brown rice, corn, and buckwheat, respectively, while the steam-cooked released about 1.4, 1.9, and 2.2 g/L of reducing sugars. At the same time, about 8–9 g/L of reducing sugars were released from extruded-puffed brown rice, corn and buckwheat. Compared to native and steam-cooked cereals, the extruded-puffed cereals greatly increased the initial hydrolysis rate. The initial hydrolysis rate of extruded-puffed brown rice, corn and buckwheat increased at least 29-, 6.2-, and 16.9-fold greater than that of native, and at least 5.6-, 4.1-, and 3.4-fold than that of steam cooked. This is because the dense structure of cereals was broken during the extrusion to form the porous structure of extrudates. The extrudates have higher surface area and degree of gelatinization for rapid digestion by amylase. As previously reported, smaller granule and irregular shaped-starch granules have greater susceptibility to be digested by amylases (Kitahara et al., 2005, Noda et al., 2008). In addition to the shape and size of granules, several factors affect the enzyme's access to the substrates and the release of hydrolysates, such as the granule integrity, crystallinity, porosity, amylose-to-amylopectin ratio, structural inhomogeneities, phosphate content, proteins, and lipids on the surface of starch granules (Blazek and Gilbert, 2010, Fan et al., 2021). Moreover, the extruded-puffed cereals have a great advantage in the time to complete hydrolysis. As shown in Fig. 2, the amount of reducing sugars released from all extruded-puffed cereals leveled off after 1 h of hydrolysis. Compared to the native or steam-cooked cereals, the completion of enzymatic hydrolysis needs more than 24 h (Blazek and Gilbert, 2010, Kunamneni and Singh, 2005). The highest reducing sugar content of extruded-puffed rice, corn, and buckwheat after hydrolysis for 3 h was ∼ 9.8 g/L. The results exhibited that the extruded-puffed cereals had a high hydrolysis conversion due to the main hydrolysis product being maltose, which was equivalent to a reducing sugar. Interestingly, although extruded-puffed buckwheat had higher bulk density and lower expansion ratio, the enzymatic hydrolysis of extruded-puffed buckwheat was not inferior to that of extruded-puffed rice or corn. This result showed that the degree of gelatinization might play an important role on amylase digestion. It has been reported that the amylopectin is significantly degraded higher than the amylose after starch extrusion (Liu et al., 2021, Liu et al., 2010, Zhang et al., 2021). The degradation of amylopectin by extrusion is similar to the action of pullulanase (debranching enzyme) and thus increases enzymatic hydrolysis efficiency. Martínez, Pico, & Gómez (2016) found that the extruded maize matrices are hydrolyzed rapidly by branching enzyme and maltogenic α-amylase. Our results showed that the extruded cereals have a faster hydrolysis rate and a higher saccharification yield. Therefore, the use of extrusion technology to improve the starch saccharification has development potential.

### 3.4 Evaluation of the extrusion effect using the kinetic constants

The effect of extrusion on the enhancement of enzymatic hydrolysis was evaluated by the Michaelis–Menten equation. The initial rate of hydrolysis reaction was investigated using substrate concentrations ranging from 5 to 20 g/L at a fixed amylase concentration of 60 U. According to the Lineweaver-Burk equation, plotting the reciprocal initial reaction rate (1/v) versus the reciprocal substrate concentrations (1/[S_0_]) is shown in Fig. S1. The kinetic constants obtained from the slope and intercept of the Lineweaver-Burk plot are shown in Table 3. K_m_ represents the affinity of enzymes and substrates; smaller K_m_ means greater affinity of the enzyme and the substrate. Extruded-puffed cereals have lower K_m_ values than steam-cooked cereals, indicating that the affinity of extruded-puffed cereals and enzyme was enhanced. The V_max_ represents the maximum number of substrates which can be converted to products per unit time; this can be used to reflect the activity of enzyme (Kuo, Chen, & Chiang, 2004). The V_max_ were 0.06–0.22 g/L min^−1^ for steam-cooked cereals, but the V_max_ were 1.42 – 2.64 g/L min^−1^ for extruded-puffed cereals. The results showed that the enzyme activity was greatly increased when the extruded-puffed cereals were used as substrate. In addition, the specificity constant can be expressed as V_max_/K_m_, which can reflect both affinity and catalytic ability (Kuo, Hsiao, Chen, Hsieh, Liu, & Shieh, 2013). It was found that the V_max_/K_m_ value of extruded-puffed brown rice, corn, and buckwheat was about 26, 12, and 24 times higher than that of steam-cooked, respectively. It means that after extrusion, the cereals greatly improve the affinity with amylase and improves the efficiency of saccharification.

**Table 3:** Kinetic constants of steam-cooked and extruded-puffed cereals obtained from Lineweaver-Burk plot.

Sample		K_m_ (g/L)	V_max_ (g/L min^−1^)	V_max_/K_m_ (min^−1^)
Brown rice	Steam-cooked	69.39	0.19	0.00274
	Extruded-puffed	37.32	2.64	0.07074

Corn	Steam-cooked	84.63	0.22	0.0026
	Extruded-puffed	71.77	2.26	0.03149
Buckwheat	Steam-cooked	49.43	0.06	0.00121
	Extruded-puffed	49.16	1.42	0.02889

### 3.5 Production of high-maltose syrup

**Fig. 3:**
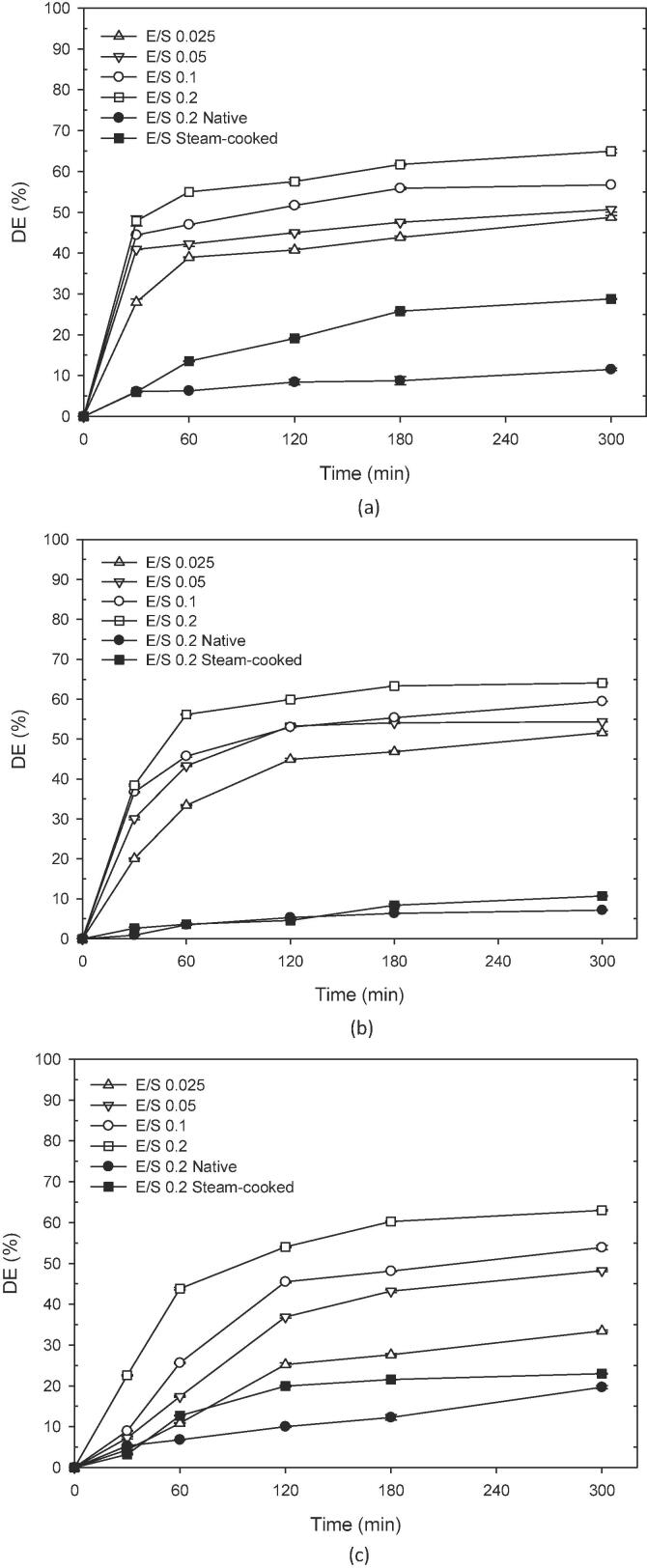
The changes in DE value during enzymatic hydrolysis of extruded-puffed (a) brown rice, (b) corn and (c) buckwheat at different E/S ratio. Hydrolysis conditions: substrate (dry basis) concentration of 200 g/L, pH 7 and 55℃.

A substrate concentration of 200 g/L (20 %, w/w) was prepared by adding water to the extruded-puffed cereals directly; the high-concentration substrate was in a viscous slurry state. The slurry was hydrolyzed by amylase at the enzyme/substrate (E/S) ratio of 0.025, 0.05, 0.1 and 0.2, respectively. Since the extruded-puffed cereals had a high initial hydrolysis rate, the viscous slurry was quickly liquefied into a solution within 10 min. The hydrolysis results are shown in Fig. 3. After 30 min, the slurry of extruded-puffed cereals was converted into a syrup solution. The hydrolysis curve leveled off and remained stable after 60 min for extruded-puffed brown rice and corn (Fig. 3a and b), but it took 180 min for extruded-puffed buckwheat (Fig. 3c). This might be related to the extruded-puffed buckwheat having higher bulk density and lower expansion ratio. The DE value of the hydrolysate increased with the E/S ratio and an E/S ratio of 0.2 showed the highest DE value of 63, 62, and 61 for extruded-puffed brown rice, corn, and buckwheat, respectively, corresponding to the maltose and glucose content determined by HPLC were 152.7 and 43.2, 156.6 and 27.7, 136.6 and 42.6 g/L. In contrast to native and steam-cooked control, the highest DE values were 8.39 and 19.11 for brown rice, 5.31 and 4.59 for corn, and 9.99 and 19.95 for buckwheat. The results indicated that extrusion processing is an effective pretreatment method for cereals to increase the efficiency of enzymatic hydrolysis and can be used to produce high-maltose syrup. Currently, the corn-based sweeteners are generally divided into four types according to the DE value. The ranges of DE value for Type I, II, III, and IV were 20–38, 38–58, 58–73, and greater than 73, respectively (Corn Refiners Association). Our results showed that by using high-concentration extruded-puffed cereals (20 %) as the substrate, the hydrolysis can be completed in 180 min. The Type II syrup can be produced from extruded-puffed cereals using E/S ratio of 0.05 to 0.1, and Type III syrup can be produced using E/S ratio of 0.1 and 0.2. The corn flour (22.0 %, w/w) was hydrolyzed by α-amylase for 3.0 h at 98 °C to obtain a syrup with DE value of 60 (Arasaratnam, Thayananthan, & Balasubramaniam, 1998). A single step high temperature hydrolysis of wheat starch using α-amylase and α-glucosidase at 90 °C obtained a syrup containing 74 % (w/w) maltose and glucose after 24 h incubation (Legin, Copinet, & Duchiron, 1998). After the cereals were extruded, the starch gelatinizes and the starch crystal structure was destroyed, which increased the reaction area and penetrating ability of amylase, improving the hydrolysis rate and digestion degree of starch, and the affinity of amylase, making extruded-puffed cereals hydrolysis more quickly and reducing the reaction time. The hydrolysis time of 180 min and reaction temperature of 50°are sufficient for extruded-puffed cereals, which greatly saves the energy consumption for enzymatic syrup production.

## 4 Conclusions

This study focused on the effect of extrusion puffing process on the physical and chemical properties of cereals and used the extrudates for amylase hydrolysis and saccharification to produce high-maltose syrup. Because of high temperature, high pressure, and mechanical shearing, extrusion more thoroughly gelatinized starch to improve enzymatic hydrolysis of starch. Gelatinization degree and FTIR-ATR results showed that extrusion puffing disrupted the crystalline structure of starch in cereal and produced a highly amorphous structure. Extruded-puffed cereals significantly improves the efficiency and yield of hydrolysis, as shown by kinetic parameters. The enzymatic hydrolysis of extruded-puffed cereals had higher saccharification conversion and shorter reaction time, indicating that the extrusion puffing treatment was suitable for the production syrup from starch-based cereals. Traditionally, syrup production is a high-temperature hydrolysis process; using extruded-puffed cereals as raw material to produce syrup has the advantages of mild reaction temperature, as well as being more economical, efficient and energy-saving.

## CRediT authorship contribution statement

**Hung-I Chien:** Data curation, Formal analysis, Investigation, Writing – original draft. **Yung-Hsiang Tsai:** Supervision. **Hui-Min David Wang:** Resources. **Cheng-Di Dong:** Supervision. **Chun-Yung Huang:** Conceptualization, Writing – review & editing. **Chia-Hung Kuo:** Conceptualization, Methodology, Supervision, Writing – original draft.

## Declaration of Competing Interest

The authors declare that they have no known competing financial interests or personal relationships that could have appeared to influence the work reported in this paper.

## Data Availability

Data will be made available on request.
