# Supplementary material for: Extrusion puffing pretreated cereals for rapid production of high-maltose syrup
Source: Food Chem X. 2022 Sep 16;15:100445. doi: 10.1016/j.fochx.2022.100445 (PMC9532787; doi:10.1016/j.fochx.2022.100445)
Supplement: Supplementary data 1 [file mmc1.docx]

(a)

(b)

(c)

**Fig. S1.** The Lineweaver–Burk plot of steam-cooked and extruded-puﬀed (a) brown rice (b) corn and (c) buckwheat.
